# Type I interferon receptor (*IFNAR2*) deficiency reveals Zika virus cytopathicity in human macrophages and microglia

**DOI:** 10.3389/fimmu.2022.1035532

**Published:** 2022-11-11

**Authors:** Aidan T. Hanrath, Catherine F. Hatton, Florian Gothe, Cathy Browne, Jane Vowles, Peter Leary, Simon J. Cockell, Sally A. Cowley, William S. James, Sophie Hambleton, Christopher J. A. Duncan

**Affiliations:** ^1^ Immunology and Inflammation Theme, Translational and Clinical Research Institute, Newcastle University, Newcastle, United Kingdom; ^2^ Department of Infection and Tropical Medicine, Royal Victoria Infirmary, Newcastle upon Tyne Hospitals NHS Foundation Trust, Newcastle, United Kingdom; ^3^ James Martin Stem Cell Facility, Sir William Dunn School of Pathology, University of Oxford, Oxford, United Kingdom; ^4^ Bioinformatics Support Unit, Newcastle University, Newcastle, United Kingdom; ^5^ School of Biomedical, Nutritional and Sports Sciences, Newcastle University, Newcastle, United Kingdom; ^6^ Department of Paediatric Immunology and Infectious Diseases, Great North Children’s Hospital, Newcastle upon Tyne Hospitals NHS Foundation Trust, Newcastle, United Kingdom

**Keywords:** Zika virus, IFNAR2 deficiency, inborn errors of immunity, macrophages, type I interferons, cell death, antiviral state, interferon-stimulated genes

## Abstract

Macrophages are key target cells of Zika virus (ZIKV) infection, implicated as a viral reservoir seeding sanctuary sites such as the central nervous system and testes. This rests on the apparent ability of macrophages to sustain ZIKV replication without experiencing cytopathic effects. ZIKV infection of macrophages triggers an innate immune response involving type I interferons (IFN-I), key antiviral cytokines that play a complex role in ZIKV pathogenesis in animal models. To investigate the functional role of the IFN-I response we generated human induced pluripotent stem cell (iPSC)-derived macrophages from a patient with complete deficiency of *IFNAR2*, the high affinity IFN-I receptor subunit. Accompanying the profound defect of IFN-I signalling in *IFNAR2* deficient iPS-macrophages we observed significantly enhanced ZIKV replication and cell death, revealing the inherent cytopathicity of ZIKV towards macrophages. These observations were recapitulated by genetic and pharmacological ablation of IFN-I signalling in control iPS-macrophages and extended to a model of iPS-microglia. Thus, the capacity of macrophages to support noncytolytic ZIKV replication depends on an equilibrium set by IFN-I, suggesting that innate antiviral responses might counterintuitively promote ZIKV persistence *via* the maintenance of tissue viral reservoirs relevant to pathogenesis.

## Introduction

Zika virus (ZIKV) is a neurotropic flavivirus of global public health importance owing to its pandemic potential (1). Effective vaccines and therapeutics for ZIKV remain elusive, although intensive efforts to develop them are ongoing. ZIKV infection is associated with pregnancy loss and neurological syndromes including microcephaly following intrauterine infection, as well as meningoencephalitis and Guillain-Barré syndrome in adults (2). Although predominantly mosquito borne, ZIKV is also capable of spreading *via* sexual transmission in semen (3). Important uncertainties concerning ZIKV pathogenesis remain, including how it accesses the central nervous system (CNS) of the developing foetus to cause microcephaly and what defines protective versus pathogenic immune responses. Addressing these questions should identify therapeutic targets and inform protective strategies to limit transmission (4).

Macrophages, immune cells that play an essential role in vertebrate host defence against microbes and in tissue repair and homeostasis (5), have been implicated in several aspects of ZIKV pathogenesis. Macrophages are key target cells of ZIKV and have been implicated both as a cellular reservoir (6), and as a vector in transmitting ZIKV to sanctuary sites such as the CNS, testes and placenta (7–10). This mechanism of viral subversion appears to rely on the ability of macrophages to support ZIKV replication without being susceptible to cytopathic effects (7–9), contrasting with other cell types such as neural progenitor cells (11–13) and epithelial cells (14). Other important viral pathogens, such as HIV, exploit a similar strategy of noncytolytic macrophage replication to form long-lived reservoirs that aid transmission to other permissive cell types (15, 16) and facilitate pathogenesis (17). ZIKV infection of human monocyte-derived macrophages is associated with induction of a robust innate immune response, dominated by type I interferons (IFN-I) (18) and proinflammatory cytokines (8, 19, 20). This appears to contrast with human monocytes (21, 22) and other myeloid cells such as dendritic cells (23, 24), which reportedly fail to induce IFNs upon ZIKV infection. Given that macrophages are permissive and ZIKV deploys multiple strategies to evade IFN-I responses (4, 25), there remains uncertainty about the functional role played by IFN-I.

Type I interferons (IFN-I) are essential to human antiviral immunity (26, 27) and patients deficient in the IFN-I receptor components *IFNAR1* or *IFNAR2* are susceptible to severe viral disease following exposure to both live-attenuated viral vaccines and wild-type viruses (26–33). Signalling through IFNAR activates a JAK-STAT signalling pathway culminating in the expression of hundreds of interferon-stimulated genes (ISGs) that govern its antiviral properties (27). Yet IFN-I plays a poorly defined and apparently complex role in ZIKV pathogenesis (4). Like other human viral pathogens, ZIKV has evolved multiple strategies to subvert IFN-I restriction in human cells (4), most notably through degradation of STAT2 by the ZIKV NS5 protein (4, 25, 34). Failure to bind murine STAT2 limits ZIKV host range (35) and prior to the development of transgenic mice encoding human *STAT2* (35), most *in vivo* studies were undertaken in *Ifnar1* deficient mice, preventing formal assessment of the confrontation between ZIKV and the IFN-I system. Whilst several ISGs have been identified that are capable of restricting ZIKV in an exogenous expression context (36–38), and pre-treatment with recombinant IFN-I provokes an antiviral state capable of restricting ZIKV infection (39), studies in human cells have yielded inconsistent findings concerning whether ZIKV induces an IFN-I response (4, 7–9, 14, 22–25, 40, 41). Adding complexity to this issue, IFN-I signalling contributed to pregnancy loss in an immunocompetent mouse model (42), suggesting that IFN-I may also be pathogenic in certain situations. To our knowledge, no study has hitherto assessed the functional impact of endogenous IFN-I responses in pathogenically relevant human cells. Progress has been in part limited by the resistance of primary human target cells such as macrophages to genetic manipulation.

Here we assess the role of IFN-I in macrophages derived from human induced pluripotent stem cells (iPSC), a valid model of yolk-sac derived, MYB-independent tissue macrophages (43–45). Generating iPSC from a patient deficient in the high-affinity type I IFN receptor subunit *IFNAR2 *(26), we identify a critical role for autocrine and paracrine IFN-I signalling in controlling ZIKV replication and protecting macrophages from ZIKV-mediated cell death. These findings were recapitulated by genetic and pharmacological ablation of IFN-I signalling in control iPS-macrophages and were also observed in a model of iPS-derived microglia (46), indicating that robust IFN-I immunity underlies the apparent resistance of tissue macrophages to ZIKV-mediated cytotoxicity and may sustain persistent infection with implications for intra-host spread and seeding of viral reservoirs.

## Results

### Generation of an *IFNAR2*-deficient iPSC macrophage model

**Figure 1:**
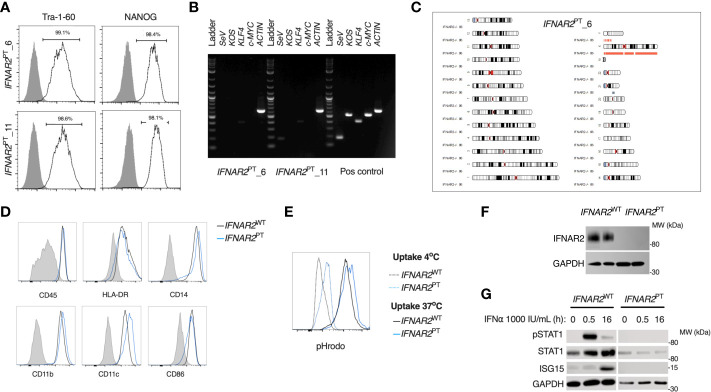
A model of *IFNAR2* deficient human iPS-macrophages (*IFNAR2* ^PT^ iPS-Mϕ). **(A)** Expression of pluripotency markers by *IFNAR2* ^PT^ iPSC clones 6 and 11 by flow cytometry.**(B)** PCR showing clearance of Sendai virus vector from *IFNAR2* ^PT^ iPSC clones 6 and 11. **(C)** Karyogram produced from SNP array showing no gross abnormalities in the previously unpublished *IFNAR2* ^PT^ iPSC clone 6. Red bars indicate loss or single copy, grey indicates loss of heterozygosity on chromosome 21 in the region of *IFNAR2* (representative of data in *IFNAR2* ^PT^ clone 11). **(D)** Expression of macrophage surface markers in *IFNAR2* ^PT^ (clone 6) and *IFNAR2* ^WT^ (WT1) iPS-Mϕ by flow cytometry, representative of repeat experiments in *IFNAR2* ^PT^ clone 11 and WT2. **(E)** Phagocytic uptake of Zymosan pHrodo particles in *IFNAR2* ^PT^ (clone 6) and *IFNAR2* ^WT^ (WT1) iPS-Mϕ, representative of repeat experiments in clone 11 and WT2. **(F)** Immunoblot of IFNAR2 and GAPDH in *IFNAR2* ^WT^ (WT2) and *IFNAR2* ^PT^ (clone 11) iPS-Mϕ, representative of repeat experiments in WT1 and clone 6. **(G)** Immunoblot of IFN-I signalling in IFNα2b (1000 IU/mL) treated *IFNAR2* ^PT^ (clone 11) and *IFNAR2* ^WT^ (WT1) iPS-Mϕ, representative of n = 3 independent experiments.

To address the impact of IFN-I signalling on macrophage responses to ZIKV infection, we derived induced pluripotent stem cells (iPSC) from dermal fibroblasts from a patient bearing a homozygous nonsense mutation of *IFNAR2 *(26), the high-affinity subunit of the IFN-I receptor (herein termed *IFNAR2* ^PT^). Following quality control assays, consisting of assessment of pluripotency marker expression by flow cytometry, elimination of Sendai vector by RT-PCR and SNP array confirmation of genome integrity (**Figures 1A–C**), we selected two clones (6 and 11) alongside two previously described healthy control lines (*IFNAR2* ^WT1-2^) (47, 48). We differentiated iPSCs to human macrophages (iPS-Mϕ) from embryoid bodies using a well-characterised, validated model (45). We quantified the expression of well-defined cell surface markers associated with macrophage differentiation including human leukocyte antigen CD45, lipopolysaccharide receptor CD14, and the major histocompatibility complex II cell surface receptor (HLA-DR) by flow cytometry (**Figure 1D**). Phagocytic function was confirmed by measuring uptake of zymosan yeast particles (pHrodo), which fluoresce in a pH-sensitive manner to demonstrate acidification in the phagolysosome (**Figure 1E**). By immunoblotting of whole cell lysates, we verified that IFNAR2 protein was absent in *IFNAR2* ^PT^ iPS-Mϕ (**Figure 1F**). Accordingly, these cells exhibited a profound defect of IFN-I signalling both at rest (as reflected in the reduction in ISG15 and STAT1 in unstimulated *IFNAR2* ^PT^ iPS-Mϕ) and upon treatment with recombinant IFNα in comparison to *IFNAR2* ^WT^ iPS-Mϕ (**Figure 1E**). Responses to IFNγ were preserved (**Figure S1**), consistent with previous findings in other cell types (26, 29, 30).

### IPS-macrophages mount a robust IFN-I response to ZIKV infection

**Figure 2:**
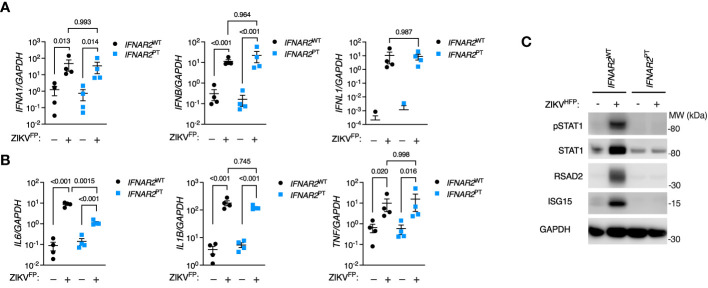
IPS-macrophages mount a robust IFN-I response to ZIKV infection. **(A)** RT-PCR quantification of *IFNA1*, *IFNB* and *IFNL1* relative to *GAPDH* (24* h*.p.i. ZIKV^FP^ MOI = 10.0, n = 4 independent experiments in *IFNAR2* ^WT^ [WT2] and *IFNAR2* ^PT^ [clone 6]). Mean ± SD, ANOVA with Sidak’s test for multiple comparisons. **(B)** RT-PCR quantification of *IL6*, *TNF* and *IL1B* relative to *GAPDH* (24* h*.p.i. ZIKV^FP^ MOI=10.0, n = 4 independent experiments in *IFNAR2* ^WT^ [WT2] and *IFNAR2* ^PT^ [clone 6]). Mean ± SD, ANOVA with Sidak’s test for multiple comparisons. **(C)** Immunoblot of pSTAT1, STAT1, RSAD2, GAPDH and ISG15 in *IFNAR2* ^WT^ (WT2) and *IFNAR2* ^PT^ (clone 11) iPS-Mϕ (48 h.p.i. ZIKV^FP^ MOI=1.0), representative of n = 3 independent experiments including *IFNAR2* ^PT^ (clone 6) and *IFNAR2* ^WT^ (WT1) iPS-Mϕ.

Initially we sought to characterise the innate immune response to infection with a clinical isolate of epidemic Asian lineage ZIKV H/FP/2013 (ZIKV^FP^) in *IFNAR2* ^PT^ and *IFNAR2* ^WT^ iPS-Mϕ. By RT-PCR analysis of cell lysates at 24 hours post-infection (h.p.i.) we observed the robust induction of *IFNA1*, *IFNB* and *IFNL1* (**Figure 2A**). Alongside the induction of *IFN* genes we observed robust expression of proinflammatory cytokines, including *IL6*, *TNF* and *IL1B* (**Figure 2B**). Only *IL6* was significantly reduced in *IFNAR2* ^PT^ iPS-Mϕ, in keeping with recent data indicating that *IL6* is induced by STAT2 and IRF9 in cooperation with NF-κB (49). Consistent with the robust induction of *IFN* genes, immunoblot analysis of lysates prepared at 48h.p.i. demonstrated tyrosine phosphorylation of STAT1, accompanied by expression of the ISG proteins RSAD2 and ISG15 in *IFNAR2* ^WT^ iPS-Mϕ, indicative of IFN signalling (**Figure 2C**). This was absent in *IFNAR2* ^PT^ iPS-Mϕ, reflecting the defect of IFN-I signalling in the latter and demonstrating that their induction by ZIKV infection was IFNAR dependent.

### Enhanced ZIKV replication in *IFNAR2*-deficient iPS-macrophages

**Figure 3:**
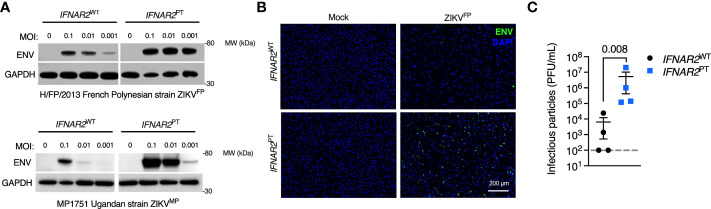
Enhanced ZIKV replication in *IFNAR2* deficient iPS-macrophages. **(A)** Immunoblot of ENV and GAPDH expression in *IFNAR2* ^WT^ (WT2) and *IFNAR2* ^PT^ (clone 11) iPS-Mϕ, 72 h.p.i. with Asian lineage (ZIKV^FP^) and African lineage (ZIKV^MP^) at the MOI demonstrated, representative of n = 3 independent experiments. **(B)** Immunofluorescence analysis of ENV expression in *IFNAR2* ^WT^ (WT2) and *IFNAR2* ^PT^ (clone 11) iPS-Mϕ (24 h.p.i. with ZIKV^FP^ MOI=1.0), representative of n = 3 independent experiments. **(C)** Plaque assay on Vero cells of ZIKV infectious particles in supernatants (48 h.p.i. ZIKV^FP^ MOI = 0.001, n = 4 independent experiments in *IFNAR2* ^WT^ [clones WT1 & WT2] and *IFNAR2* ^PT^ [clones 6 & 11]). Mean ± SD, t test.

We next sought to assess the impact of the loss of IFN-I signalling on replication of ZIKV^FP^ by analysis of viral Envelope (ENV) protein expression by immunoblot of whole cell lysates at 72 h.p.i., or by immunofluorescence staining of fixed and permeabilised cells at 24 h.p.i., alongside plaque assay on permissive Vero cells of infectious particle release in supernatants (**Figures 3A–C**). These analyses demonstrated productive ZIKV infection that was significantly enhanced in *IFNAR2* ^PT^ iPS-Mϕ across a range of multiplicities of infection (MOI). This phenotype was independent of viral origin, as it was also seen with African lineage MP1751 infection (ZIKV^MP^ **Figure 3A**). These data were consistent with a study employing JAK inhibitors in primary placental macrophages (50), demonstrating that the robust IFN-I response of macrophages is functional and acts to limit productive ZIKV replication.

### IFN-I mediates paracrine protection of macrophages

**Figure 4:**
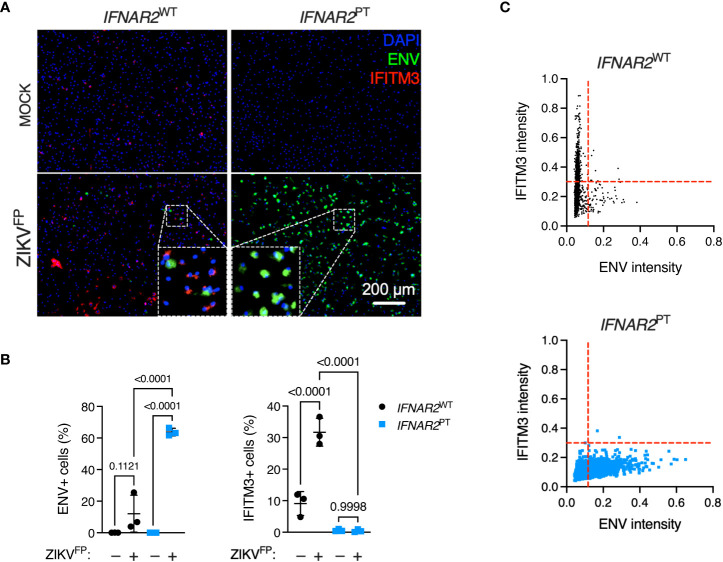
IFN-I mediates paracrine protection of iPS-macrophages. **(A)** Immunofluorescence analysis of ZIKV ENV and IFITM3 expression in *IFNAR2* ^PT^ (clone 11) and *IFNAR2* ^WT^ (WT2) iPS-Mϕ (24 h.p.i. ZIKV^FP^ MOI = 10.0). Representative images from one of three independent experiments are shown. Scale bar = 200 μm. **(B)** CellProfiler quantification of images in **(A)** showing proportion of cells expressing ZIKV ENV (left panel) or the ISG IFITM3 (right panel). Mean ± SD of n = 3 independent experiments, ANOVA with Sidak’s test for multiple comparisons. **(C)** CellProfiler analysis of single cell expression of ENV and IFITM3 in *IFNAR2* ^WT^ and *IFNAR2* ^PT^ iPS-Mϕ from images in **(A)**, n = 2,434 (WT) cells and n = 2,202 (PT) cells respectively. Red dotted lines represent gating. Representative data from one of three independent experiments.

To explore this interaction at the single cell level, we undertook immunofluorescence analysis of ZIKV ENV and ISG expression *via* immunostaining for IFITM3 in cells at 24 h.p.i. with ZIKV^FP^, infecting with MOI 10 in order to achieve detectable ENV expression in WT iPS-Mϕ (**Figure 4A**). This analysis revealed a significant increase of ENV expression in *IFNAR2* ^PT^ iPS-Mϕ (approximately 60%, compared to an average of 10-15% in *IFNAR2* ^WT^ iPS-Mϕ), accompanied by a failure of both basal and inducible IFITM3 expression in *IFNAR2* ^PT^ iPS-Mϕ (**Figure 4B**), consistent with our earlier findings. Upon ZIKV exposure of *IFNAR2* ^WT^ iPS-Mϕ, IFITM3 expressing cells were frequently seen surrounding infected cells, the latter defined by ZIKV ENV expression (see inset box, **Figure 4A**), suggesting paracrine signalling. IFITM3 positive bystander cells were not seen in *IFNAR2* ^PT^ iPS-Mϕ cultures. Using the image analysis tool CellProfiler, we compared expression of these markers to define the relationship between infection and ISG expression by genotype at the single cell level. Analysis of >2000 cells per genotype revealed that upon ZIKV infection in *IFNAR2* ^WT^ iPS-Mϕ, IFITM3 was predominantly expressed in uninfected bystander (ENV-) cells, consistent with paracrine regulation of IFITM3 expression. By contrast, *IFNAR2* ^PT^ iPS-Mϕ lacked IFITM3 expression in bystander cells, reflecting the profound loss of paracrine IFN-I response. Interestingly, in infected (ENV+) *IFNAR2* ^WT^ iPS-Mϕ, IFITM3 was largely undetectable (**Figure 4C**), suggesting that ZIKV infection prevented IFN-I signalling in infected cells, consistent with prior studies (25, 34).

### Increased vulnerability to ZIKV cytopathic effects in *IFNAR2*-deficient iPS-macrophages

**Figure 5:**
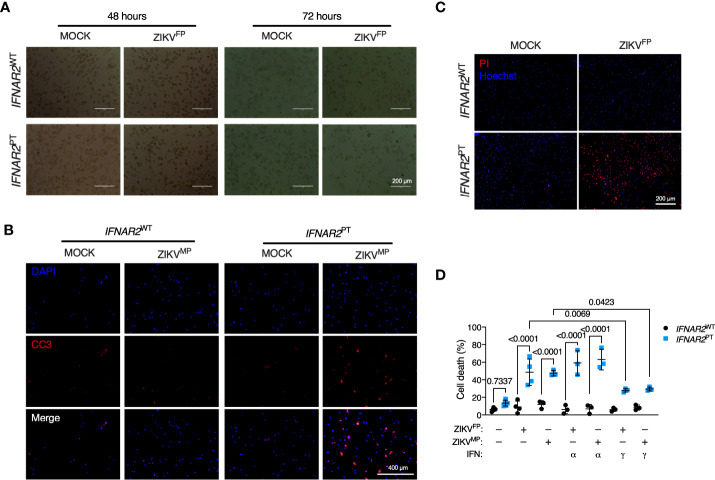
*IFNAR2*-deficient iPS-macrophages are vulnerable to ZIKV cytopathic effects. **(A)** Progressive cytopathicity in *IFNAR2* ^PT^ (clone 6) but not *IFNAR2* ^WT^ (WT2) iPS-Mϕ following infection with ZIKV^FP^ MOI = 1.0, showing morphological features of cell shrinkage, membrane blebbing and cell fragmentation. Scale bar, 200 μm. **(B)** Immunofluorescence analysis of cleaved caspase 3 (CC3) at 48 h.p.i. ZIKV^MP^ MOI = 1.0. Representative images of two independent experiments. Scale bar, 400 μm. **(C)** Immunofluorescence analysis of cell viability showing representative images of cell death at 72 h.p.i. ZIKV^FP^ MOI = 1.0 in *IFNAR2* ^PT^ (clone 11) but not *IFNAR2* ^WT^ (WT2) iPS-Mϕ, representative of n = 4 independent experiments. Scale bar, 200 μm. **(D)** CellProfiler quantification of cell viability assay in *IFNAR2* ^PT^ (clone 11) and *IFNAR2* ^WT^ (WT1 and WT2) iPS-Mϕ with or without recombinant IFNα2b or IFNγ (1000 IU/mL) pretreatment. 72 h.p.i. ZIKV^FP^ MOI = 1.0 (n = 4 independent experiments) or ZIKV^MP^ MOI = 1.0 (n = 3 independent experiments). Mean ± SD, ANOVA with Sidak’s test for multiple comparisons.

Accompanying heightened ZIKV replication in IFN-I incompetent cells, we observed morphological abnormalities suggestive of cell death, with progressive membrane blebbing and cell shrinkage from 48 h.p.i. (**Figure 5A**) accompanied by immunodetection of cleaved caspase 3 (**Figure 5B**), indicative of apoptosis and in keeping with previous reports of the mechanism of ZIKV-induced cell death in other cell types (11, 14). These data indicated that failure of IFN-I mediated control rendered *IFNAR2* ^PT^ iPS-Mϕ vulnerable to cytopathic effects (CPE), contrasting with the resistance of WT macrophages to ZIKV CPE observed by ourselves and previously reported (7–9). We confirmed this using an imaging based live-cell viability assay which showed cell death in approximately 60% of *IFNAR2* ^PT^ iPS-Mϕ at 72 h.p.i. (**Figure 5C**). Quantification of these data using CellProfiler demonstrated that ZIKV CPE was significantly enhanced in *IFNAR2* ^PT^ but not *IFNAR2* ^WT^ iPS-Mϕ, a phenotype that was independent of viral lineage, as it was observed equally following ZIKV^FP^ and ZIKV^MP^ infections (**Figure 5D**). Prior treatment with IFNα2b (1000 IU/mL) was unable to protect *IFNAR2* ^PT^ iPS-Mϕ from cytopathic effects, consistent with their defect of endogenous IFN-I signalling. By contrast, treatment of *IFNAR2* ^PT^ iPS-Mϕ with IFNγ partially rescued ZIKV-induced cell death (**Figure 5D**), in keeping with their intact response to IFNγ (**Figure S1**), indicating that *IFNAR2* ^PT^ iPS-Mϕ were not globally immunodeficient.

### Pharmacologic or genetic ablation of IFN-I signalling recapitulates iPS-macrophage vulnerability

**Figure 6:**
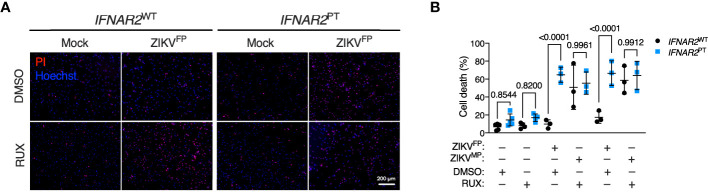
JAK inhibition recapitulates ZIKV cytopathicity in wild-type iPS-macrophages. **(A)** Immunofluorescence analysis of cell viability showing representative images of cell death at 72 h.p.i. in *IFNAR2* ^WT^ (WT1) and *IFNAR2* ^PT^ (clone 11) iPS-Mϕ treated with RUX (10 uM) or DMSO control (ZIKV^FP^ MOI = 1.0, representative images of n = 4 independent experiments). Scale bar = 200 μm. PI = propidium iodide. **(B)** CellProfiler quantification of cell viability assay in *IFNAR2* ^PT^ (clone 11) and *IFNAR2* ^WT^ (WT1 and WT2) iPS-Mϕ treated with RUX (10 uM) or DMSO control (72 h.p.i. ZIKV^FP^ MOI = 1.0, n = 4 independent experiments and ZIKV^MP^ MOI = 1.0, n = 3 independent experiments). Mean ± SD, ANOVA with Sidak’s test for multiple comparisons. .

These observations implied that defective IFN-I signalling accounted for heightened ZIKV replication and vulnerability to cell death in *IFNAR2* ^PT^ iPS-Mϕ. However we could not rule out the possibility that these findings related to another aspect of the genetic background of this *IFNAR2* deficient patient. To validate our findings, we adopted two complementary approaches of IFNAR blockade to determine whether this phenotype could be recapitulated in control iPS-Mϕ. First, we used the JAK inhibitor ruxolitinib (RUX) at a dose (10 μM) previously shown to ablate IFN-I signalling (51). Treatment of *IFNAR2* ^WT^ iPS-Mϕ with RUX for 16 h prior to infection led to a significant enhancement of ZIKV-induced cell death compared to vehicle (DMSO) treatment (**Figure 6**), reproducing our previous observations in *IFNAR2* ^PT^ iPS-Mϕ and suggesting that loss of the inducible signalling response to ZIKV infection is the major mechanism by which *IFNAR2* deficiency contributes to macrophage susceptibility. By contrast, treatment of *IFNAR2* ^WT^ iPS-Mϕ with RUX prior to infection did not further enhance cell death, indicating that other IFN-I independent, JAK dependent signalling pathways (including IFNλs) made little contribution to protection against cytopathic effects.

**Figure 7:**
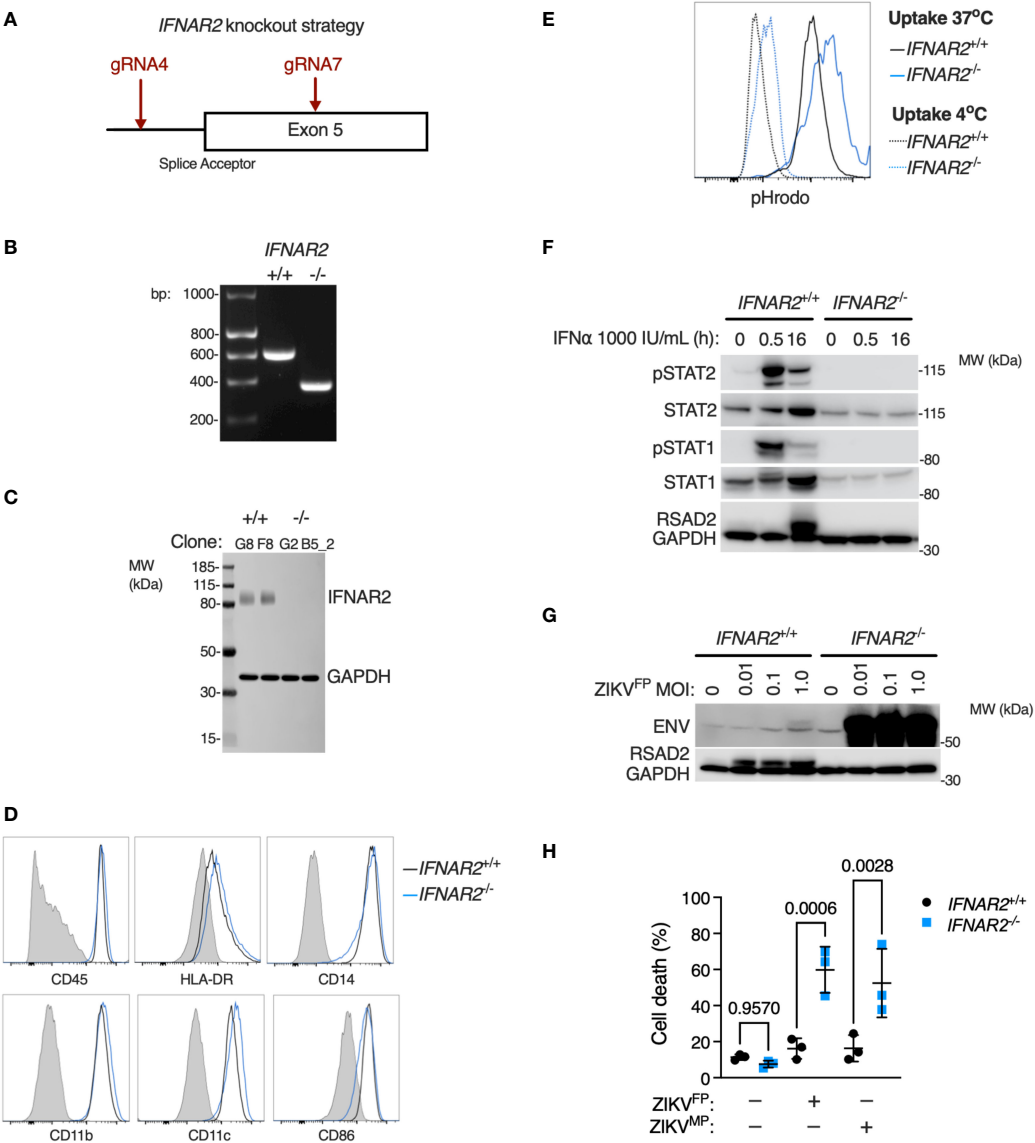
*IFNAR2* knockout in wild-type iPS-macrophages recapitulates heightened ZIKV replication and cytopathicity. **(A)** CRISPR/Cas9 guide design. **(B)** PCR of *IFNAR2* amplicon demonstrating exon 5 splice acceptor site excision in iPSC clones (G8 and G2). Representative of experiments in clones F8 and B5_2. **(C)** Immunoblot of *IFNAR2* and GAPDH expression, demonstrating IFNAR2 ablation in iPS-Mϕ clones (G8, F8, G2, B5_2). Representative of n=2 independent experiments. **(D)** Expression of macrophage surface markers in *IFNAR2* ^-/-^ (B5_2) and *IFNAR2* ^+/+^ (F8) iPS-Mϕ by flow cytometry, representative of repeat experiments in F8/B5_2. **(E)** Phagocytic uptake of Zymosan pHrodo particles in *IFNAR2* ^-/-^ (B5_2) and *IFNAR2* ^+/+^ (F8) iPS-Mϕ by flow cytometry, representative of repeat experiments in F8/B5_2. **(F)** Immunoblot of IFN-I signalling in IFNα2b (1000 IU/mL) treated *IFNAR2* ^-/-^ (B5_2) and *IFNAR2* ^+/+^ (F8) iPS-Mϕ, representative of n = 3 independent experiments. **(G)** Immunoblot of ENV, RSAD2 and GAPDH expression in *IFNAR2* ^-/-^ (B5_2) and *IFNAR2* ^+/+^ (F8) iPS-Mϕ, 72 h.p.i. post infection, representative of n=3 independent experiments. **(H)** CellProfiler quantification of cell viability assay in *IFNAR2* ^-/-^ (G2, B5_2) and isogenic *IFNAR2* ^+/+^ (G8, F8) iPS-Mϕ (72 h.p.i. ZIKV^FP^ or ZIKV^MP^ MOI = 1.0, n = 3 independent experiments). Mean ± SD, ANOVA with Sidak’s test for multiple comparisons.

Given that JAK inhibition can impact a broad range of IFN and cytokine signalling pathways we sought to provide conclusive genetic evidence of the relevance of IFN-I to this phenotype, using CRISPR/Cas9 to delete *IFNAR2* in WT1 and WT2 control iPSC lines (**Figure 7A**), selecting *IFNAR2* ^-/-^ and isogenic *IFNAR2* ^+/+^ single cell iPSC clones for differentiation into iPS-Mϕ. *IFNAR2* knockout was verified by PCR (**Figure 7B**) and confirmed by immunoblotting of *IFNAR2* ^-/-^ iPS-Mϕ lysates (**Figure 7C**). Macrophage phenotyping was undertaken as previously described for *IFNAR2* ^PT^ iPS-Mϕ and, as before, was unaffected by *IFNAR2* expression status (**Figures 7D, E**). In comparison to *IFNAR2* ^+/+^ iPS-Mϕ, *IFNAR2* ^-/-^ iPS-Mϕ exhibited a profound defect of IFN-I signalling both at rest - reflected in the reduction in basal STAT1 and STAT2 in unstimulated lysates - and upon treatment with recombinant IFNα, where phosphorylation of the signalling intermediates STAT1 and STAT2 and induction of RSAD2 protein was absent (**Figure 7F**). This mirrored our earlier observations in *IFNAR2* ^PT^ iPS-Mϕ. Consequently, infection of *IFNAR2* ^-/-^ iPS-Mϕ with ZIKV^FP^ recapitulated the enhanced permissiveness to infection and CPE previously observed in *IFNAR2* ^PT^ iPS-Mϕ (**Figures 7G, H**), confirming the association of the observed phenotypes with defective IFNAR signalling.

### Genome-wide transcriptional profiling of *IFNAR2* ^-/-^ and isogenic *IFNAR2* ^+/+^ iPS-macrophages

**Figure 8:**
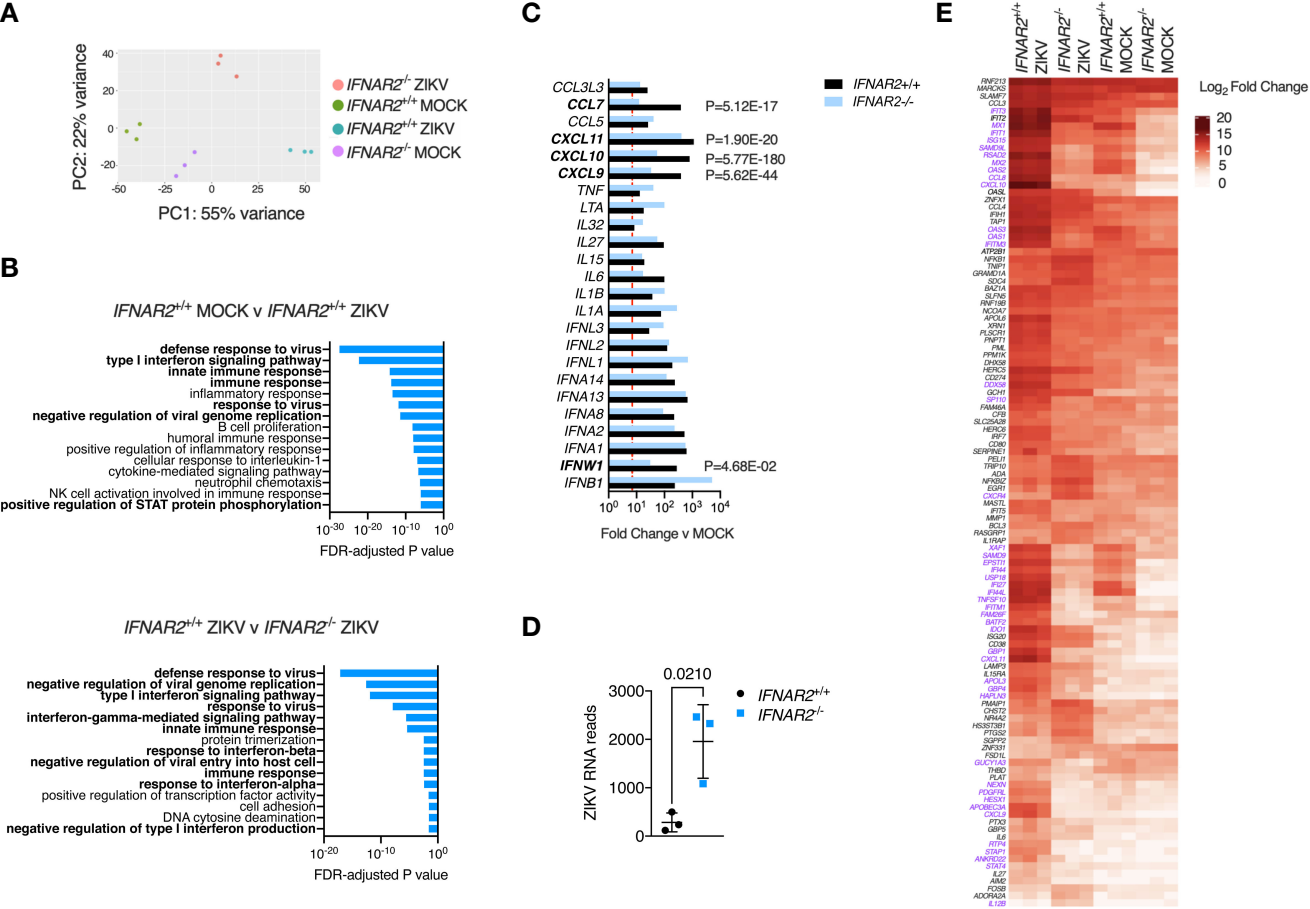
IFN-I signalling dominates the transcriptional response to ZIKV infection. **(A)** Principal component analysis of RNA-seq data (24 h.p.i. ZIKV^FP^ MOI = 1.0, n=3 biological replicates in isogenic *IFNAR2* ^-/-^ [B5_2] and *IFNAR2* ^+/+^ [F8] iPS-Mϕ). **(B)** Gene ontology analysis (FDR<5%) of RNA-seq data, comparing mock v infected *IFNAR2* ^+/+^ iPS-Mϕ (top) and *IFNAR2* ^-/-^ v *IFNAR2* ^+/+^ iPS-Mϕ (bottom). Selected pathways highlighted in bold. **(C)** Significantly differentially expressed DE IFN, chemokine and cytokine genes, comparing mock v ZIKV exposed conditions for *IFNAR2* ^+/+^ (black bars) or *IFNAR2* ^-/-^ genotype (blue bars). Genes not reaching the DE threshold (Log_2_ FC ≥ 3, FDR < 5%) in both genotypes are not displayed. Red dotted line represents FC threshold (Log_2_ FC ≥ 3). FDR-adjusted P values are also included for significantly DE genes (in bold) comparing *IFNAR2* ^-/-^ and *IFNAR2* ^+/+^ ZIKV exposed datasets. **(D)** Aligned ZIKV reads from data in **(A)**, mean ± SD, t test. **(E)** Heatmap displaying expression of annotated macrophage-specific ISGs. Colour intensity reflects Log_2_ FC. Significantly DE genes (Log_2_ FC ≥ 3, FDR < 5%) are shown in purple text for the comparison of *IFNAR2* ^-/-^ and *IFNAR2* ^+/+^ ZIKV exposed datasets.

An advantage of the CRISPR/Cas9 approach is that it provides *IFNAR2* ^-/-^ and *IFNAR2* ^+/+^ iPS-Mϕ on an isogenic background, overcoming donor-donor variation in gene expression that can potentially confound differential expression (DE) analysis (52). To explore the global transcriptional response to ZIKV in an unbiased manner, we undertook RNA-sequencing of libraries prepared from ZIKV^FP^ and mock-infected *IFNAR2* ^-/-^ and isogenic *IFNAR2* ^+/+^ iPS-Mϕ at 24 h.p.i (clones B5_ 2 and F8 respectively). Principal component and pathway analyses revealed the dominant contribution of IFNAR signalling to the ZIKV response, consistent with our prior findings, and also revealed substantial differences in the transcriptome of uninfected *IFNAR2* ^-/-^ and isogenic *IFNAR2* ^+/+^ iPS-Mϕ, consistent with the loss of ‘tonic’ IFN-I signalling in *IFNAR2* ^-/-^ iPS-Mϕ (**Figures 8A, B**). Of 492 genes significantly DE in *IFNAR2* ^+/+^ compared to *IFNAR2* ^-/-^ iPS-Mϕ upon ZIKV infection, 467 (95%) were ISGs based on interrogation of the gene lists in the Interferome database (53) (http://interferome.its.monash.edu.au/interferome). Consistent with our prior findings, a broad range of *IFN*, chemokine and cytokine genes were induced upon ZIKV infection in both *IFNAR2* ^-/-^ and isogenic *IFNAR2* ^+/+^ iPS-Mϕ, with *IFNs* predominating (**Figure 8C**). ZIKV reads were also significantly enriched in *IFNAR2* ^-/-^ iPS-Mϕ (**Figure 8D**) at this relatively early timepoint, consistent with the findings of our earlier analyses. To complement this analysis, we visualised the expression of a subset of context-specific ISGs induced in human macrophages by IFN-I treatment (54), annotating significantly DE genes between *IFNAR2* ^-/-^ and isogenic *IFNAR2* ^+/+^ iPS-Mϕ upon ZIKV exposure (**Figure 8E**). This emphasised the profound alteration to the transcriptome of *IFNAR2* ^-/-^ iPS-Mϕ at rest and upon ZIKV infection. It also revealed the more modest induction of a subset of ISGs in *IFNAR2* ^-/-^ iPS-Mϕ, presumably secondary to IFNAR-independent induction of ISGs by IRF3 (55). Regardless of the mechanism, IFNAR-independent ISG induction was insufficient to have a functional impact on antiviral protection under the experimental conditions studied.

### Enhanced ZIKV infection and CPE in *IFNAR2*-deficient iPS-microglia-like cells

**Figure 9:**
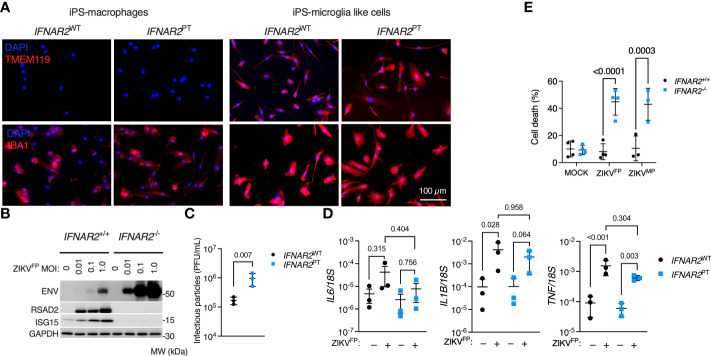
Enhanced ZIKV infection and CPE in *IFNAR2*-deficient iPS-microglia-like cells. **(A)** Immunofluorescence analysis of microglial markers IBA1 and TMEM119 in *IFNAR2* ^PT^ (PT clone 11) and *IFNAR2* ^WT^ (WT2) iPS-Mϕ and iPS-microglia-like cells (iPS-MGLs), representative of repeat experiments in G2, B5_2, G8 and F8 lines. Scale bar = 100 μm. **(B)** Immunoblot of ENV, RSAD2, ISG15, GAPDH in ZIKV^FP^-infected *IFNAR2* ^+/+^ (F8) and *IFNAR2* ^-/-^ (B5_2) iPS-MGLs at 72 h.p.i., representative of n = 3 independent experiments in *IFNAR2* ^PT^ [clones 6 and 11] and *IFNAR2* ^WT^ [WT2]). **(C)** Plaque assay on Vero cells of ZIKV infectious particles in supernatants (48 h.p.i. ZIKVFP MOI = 1, n = 3 independent experiments in *IFNAR2* ^WT^ (WT2) and *IFNAR2* ^PT^ (clone 6) iPS-microglia-like cells. Mean ± SD, t test. **(D)** RT-PCR quantification of *IL6*, *IL1B* and *TNF* relative to *18S* (24* h*.p.i. ZIKV^FP^ MOI = 1, n = 3 independent experiments in *IFNAR2* ^WT^ [WT2] and *IFNAR2* ^PT^ [clones 6 and 11]) microglia-like cells. Mean ± SD, ANOVA with Sidak’s test for multiple comparisons. **(E)** Cell viability assay (72 h.p.i. ZIKV^FP^ MOI = 1.0 [n = 4 independent experiments] or ZIKV^MP^ MOI = 1.0 [n = 3 independent experiments]) in *IFNAR2* ^-/-^ (B5_2), *IFNAR2* ^PT^ (clone 6), *IFNAR2* ^+/+^ (F8) and *IFNAR2* ^PT^ (WT2) iPS-MGLs. Mean ± SD, ANOVA with Sidak’s test for multiple comparisons.

According to our current understanding, human microglia originate from yolk-sac progenitors which enter the CNS early in development, prior to closure of the blood-brain barrier, and are maintained there by local proliferation (56). As the main brain-resident macrophage population, microglia have key roles in homeostasis and pathogen defence (56). However, these cells have also been implicated as a ‘Trojan horse’, potentially transmitting ZIKV to the developing brain during vertical transmission in early pregnancy and contributing to the development of microcephaly (9, 10, 57). To understand the relevance of our observations to microglia, we employed a validated method for differentiating microglia-like cells from iPSC (iPS-MGL) (46). We differentiated *IFNAR2* ^PT^ and *IFNAR2* ^WT^ iPSC, in addition to *IFNAR2* ^-/-^ and isogenic *IFNAR2* ^+/+^ iPSC, to iPS-MGL, confirming by immunofluorescence analysis expression of the microglial marker TMEM119 (58, 59), alongside increased expression of IBA1 compared to iPS-Mϕ (**Figure 9A**). Upon infection with ZIKV^FP^ and ZIKV^MP^ we observed the same phenotypes in iPS-MGL that were previously seen in IFNAR2-deficient iPS-Mϕ, including a robust pro-inflammatory response, significantly heightened ZIKV replication, increased infectious particle release and vulnerability to CPE (**Figures 9B–E**), indicating that IFN-I mediated restraint of ZIKV replication and cytopathicity is also key to the response of CNS-resident human microglia to ZIKV infection.

## Discussion

Many successful human pathogens have evolved sophisticated mechanisms to subvert tissue macrophages - exploiting this cellular niche for survival and/or transmission (14). Here we employed genetically tractable models of human macrophages and microglia derived from wild-type and *IFNAR2* deficient iPSCs to interrogate interactions of ZIKV, an RNA viral pathogen of global public health importance, with the innate immune system of macrophages. We observe that the ability of wild-type iPS-macrophages to resist cytopathic effects of ZIKV depends on robust autocrine and paracrine IFN-I signalling that acts to contain viral replication and spread. ZIKV is highly human-adapted, relying on myriad methods to evade restriction by antiviral IFN signalling, especially *via* the targeting of human (but not mouse) STAT2 protein (4, 25, 34). Our data resolve an open question concerning the extent to which human endogenous IFN-I responses are capable of restricting ZIKV replication in the face of these viral countermeasures, providing a framework in which to explore host susceptibility to ZIKV disease and identify targets for host-directed antiviral therapy.

Although the question of the functional role of IFN-I in clinically relevant ZIKV target cells had not been addressed directly, there is evidence that IFN-I participates in ZIKV resistance of target cells. For instance, genetic ablation of *AXL* attenuated ZIKV replication in an astrocytic cell line, seemingly by enhancing endogenous IFN-I signalling (40). AXL is a recognised negative regulator of IFNAR, indicating that loss of AXL may have promoted IFN-I signalling. Similarly, deletion of the ISG *ISG15* reduced ZIKV replication in iPS-derived neural progenitor cells (iPS-NPCs) (41). Whilst this result might at first glance appear paradoxical, ISG15 has divergent species-specific function, acting in mouse as an antiviral effector but in human as a negative regulator of IFNAR signalling (60). Consistent with these data, blockade of IFN-I signalling with JAK inhibitors or IFNAR antibodies was reported to enhance ZIKV replication in placental macrophages (50) and iPS-microglia (9). Importantly, the consequences of IFN-I inhibition for macrophage survival were not assessed.

Our data, relying on a robust genetic approach involving use of *IFNAR2* deficient patient iPSCs and validated by precise CRISPR/Cas9 *IFNAR2* knockout in control iPSCs, confirm and substantially extend this indirect evidence, showing that IFN-I is decisive in controlling ZIKV replication despite extensive viral countermeasures (4), in turn protecting iPS-macrophages and iPS-microglia from profound cytopathicity. Thus macrophage lineage cells are not as a population intrinsically resistant to cytopathic effects of ZIKV, as is widely reported (7–9) but instead mount a robust paracrine IFN-I response that restrains viral replication and maintains viability of the majority of bystander cells. This IFN-I restriction presumably explains why experimental manipulation, such as the use of flavivirus immune serum to enhance macrophage ZIKV uptake (61), is necessary to enhance infection efficiency of primary monocyte-derived macrophages (18).

The single cell imaging analyses presented here indicate that IFN-I mediated protection of wild-type iPS-macrophages derives largely from paracrine IFN-I signalling, suggesting that IFN-I produced by macrophages might also confer protection on neighbouring stromal cell types present in tissues *in natura*. This idea that macrophages might act in a protective capacity as antiviral sentinels promoting IFN-mediated defence of neighbouring cells is supported by recent findings in animal models (62, 63). However, our data are also consistent with a model in which innate antiviral IFN responses counterintuitively promote ZIKV persistence by preventing macrophage depletion, facilitating the maintenance of macrophage reservoirs in sanctuary sites such as the brain or testes (7–10). Similar findings were reported for human fetal astrocytes, which supported long-term ZIKV replication in the face of a robust innate immune response (64). Thus our data imply a relatively complex role for IFN-I signalling in ZIKV pathogenesis, contributing on the one hand to protection of macrophages against unrestrained ZIKV replication and cytopathic effects, whilst on the other maintaining an infected reservoir in immune sanctuary sites. Examples from paramyxovirus models indicate that innate immune responses may contribute to RNA virus persistence (65, 66). The specific contribution of IFN-I responses to viral persistence in macrophages in other relevant families, such as the *Retroviridae* and *Togaviridae*, will be an important line of investigation (67).

Our focus on iPS-macrophages and iPS-microglia is the principal limitation of this study. It is likely that co-culture and/or organoid models will be needed in future studies to model the collective impact of microglial IFN-I signalling on viral behaviour in neighbouring CNS target cells such as astrocytes and neural progenitor cells. Nevertheless, the use of this system provided unambiguous genetic evidence by enabling the analysis of macrophages derived from patients with ultra-rare genetic disorders where cellular material is limited and simultaneously provided a means of overcoming the resistance of macrophages to genetic manipulation by CRISPR/Cas9. Our studies also employed the two major ZIKV lineages (African and Asian), lending generalisability to the findings.

In summary, the data presented here show that human macrophages and microglia-like cells respond to ZIKV infection with robust paracrine IFN-I signalling that restricts viral replication and promotes cell survival in the face of ongoing infection. These data establish the functional role of IFN-I in control of ZIKV replication in a disease-relevant cell type and shed light on the complex role played by macrophages in ZIKV pathogenesis. Given that microcephaly and encephalitis are relatively rare outcomes of ZIKV infection, an important question for future studies will be the extent to which defects in host IFN-I immunity [via genetic lesions (26–33) or neutralising IFN-I autoantibodies (68)] might contribute to the development of CNS complications of ZIKV disease. Another question for future studies is whether the mode of IFN-I dependent resistance to viral cytopathic effects we report applies more generally to RNA viruses that target and exploit macrophages (5).

## Methods

### iPSC reprogramming and culture

Human induced pluripotent stem cells (iPSC) were reprogrammed from skin dermal fibroblasts from a patient carrying a homozygous nonsense variant in *IFNAR2* (c.A311del, p.Gly104fs110X, termed *IFNAR2* ^PT^). Written informed parental consent was provided (Newcastle and North Tyne Research Ethics Committee Ref: 16/NE/0002). Briefly, fibroblasts were reprogrammed at passage 3-5, using Cytotune 2 Sendai virus reprogramming kit (Invitrogen) according to the manufacturer’s instructions, with modifications as described (47). QC assays on bulk banked stocks (at or near to passage 10) included flow cytometry assessment of pluripotency marker expression, RT-PCR to check clearance of CytoTune vectors and Illumina SNP array to check genome integrity and identity/tracking back to the source fibroblasts (**Figures S1A–C**). Clones 6 and 11 were used for experiments.

The *IFNAR2* ^WT^ control iPSC lines SFC841-03-01 (48) (WT1, Ref: STBCi044-A) and SFC856-03-04 (47) (WT2, Human Pluripotent Stem Cell Registry Ref: STBCi063-A) were previously generated using the same methodology, at the James Martin Stem Cell Facility at the University of Oxford. These lines are available from the European Bank for Induced pluripotent Stem Cells (EBISC). iPSCs were cultured feeder-free on Geltrex (Gibco) coated plates in mTeSR1 (Stem Cell Technologies, Canada) media, exchanged daily. Cultures were passaged using 0.5 mM EDTA (Gibco). Cryopreserved master stocks of iPSC were used and passaged no more than three times prior to differentiation to macrophages or microglia.

### *IFNAR2* knockout (KO) using CRISPR/Cas9

The control iPSC lines SFC856-03-04 and SFC841-03-01 were used for gene editing. Two guide RNAs (gRNAs) were used to target the *IFNAR2* exon 5 splice acceptor (reference transcript: NM_001289125.3). This was predicted to cause out of frame skipping of exon 5 from all processed *IFNAR2* transcripts, leading to nonsense mediated RNA decay and absence of IFNAR2 protein expression. The guide RNA (gRNA) sequences were: CATTTTCAATAAGATGGTTG (gRNA4) and ACCGTCCTAGAAGGATTCAG (gRNA7). These gRNAs were purchased from Integrated DNA Technologies (IDT, USA) and were complexed to tracRNA, before mixing with 1.5 μg Alt-R HiFi Cas9 Nuclease (IDT, USA) to form a ribonucleoprotein (RNP) complex, according to manufacturer’s instructions. The gRNA : Cas9 RNP complex was transfected into 2 x 10^5^ iPSCs in single-cell suspension by electroporation (Neon transfection system, ThermoFisher Scientific) in 10 μL using the ‘HiTrans’ settings: 1400 v, 20 ms width, 1 pulse. iPSC were treated with 10 μL RHO kinase inhibitor Y-27632 (AbCam Biochemicals) for 3 h prior to transfection. After 3 d iPSC were single-cell plated onto irradiated mouse embryonic fibroblast feeders. Single-cell colonies were subsequently picked, expanded under feeder-free conditions and screened by PCR gel electrophoresis (primers available on request). One *IFNAR2* ^-/-^ and isogenic *IFNAR2* ^+/+^ pair was selected from each parental line for further validation by PCR (**Figure 7B**) and capillary sequencing (not shown). SFC856-03-04 clones were B5_2 (*IFNAR2* ^-/-^) and F8 (*IFNAR2* ^+/+^); SFC841-03-01 clones were G2 (*IFNAR2* ^-/-^) and G8 (*IFNAR2* ^+/+^). IFNAR2 expression was examined by immunoblot.

### Cells, cytokines and inhibitors

Vero cells were cultured in complete Dulbecco’s modified Eagle’s medium (DMEM; Gibco) supplemented with 10% foetal calf serum (FCS; Gibco), 1% penicillin/streptomycin (Gibco) and 1% L-glutamine (Gibco). All cells were incubated in a humidified atmosphere with 5% CO_2_ at 37°C. Cytokines/inhibitors were used at the following concentrations: human recombinant IFN-α2b (1000 IU/ml; Intron A, Schering-Plough, USA); IFN-γ (1000 IU/ml; Immunikin, Boehringer Ingelheim, Germany) and Ruxolitinib (10 μM; Calbiochem, USA).

### Macrophage and microglia differentiation

iPSCs were differentiated to macrophage precursor cells *via* embryoid bodies (EBs) using AggreWells (Stem Cell Technologies) using a published methodology with no alterations (45). Media for embryoid body generation consisted of mTeSR with 50 mg/mL BMP4, 50 mg/mL VEGF and 20 mg/mL SCF. Approx. 300 EBs were split between two T175 flasks in 15 mL of medium per flask. ‘Factory’ medium comprised XVIVO15 (Lonza, Basel, Switzerland) with 100 ng/mL recombinant human M-CSF (Gibco), 25 ng/mL recombinant human IL-3 (Gibco), 2 nM GlutaMAX (Gibco), 50 μM 2-mercaptoethanol and 100 units/mL penicillin with 100 µg/mL streptomycin (Gibco). Precursor cells were harvested and plated into final experimental format for final differentiation to either iPS-Mϕ in macrophage differentiation medium (XVIVO15 with 100ng/mL recombinant human M-CSF (Gibco), 2 mM GlutaMAX (Gibco) and 100 units/mL penicillin with 100 µg/mL streptomycin) for 7 days prior to use, or iPS-microglia-like cells (iPS-MGLs) in microglia differentiation medium (Advanced DMEM/F12 + N2 Supplement (Gibco) with 100 ng/mL recombinant human IL-34 (PeproTech), 10 ng/mL recombinant human GM-CSF (Gibco), 2 mM Glutamax, 50 μM 2-mercaptoethanol and 100 units/mL penicillin with 100 µg/mL streptomycin) for 14 days, changing medium twice weekly, prior to use in experiments.

### ZIKV propagation and infection

Two strains of ZIKV were used in this study, the epidemic Asian lineage strain H/FP/2013 (patient isolate, French Polynesia, 2013, herein ZIKV^FP^) was obtained from the European Virus Archive (Ref: 001v-EVA1545) and the African lineage strain MP1751 (mosquito isolate, Uganda, 1962, herein ZIKV^MP^) was obtained from the UK National Collection of Pathogenic Viruses (Ref: 1308258v). Viral stocks were propagated at low MOI in Vero cells (obtained from Professor R Randall, St Andrew’s University), and viral titre determined by serial dilution and standard plaque assay. Stocks were aliquoted, clarified by centrifugation and clarified supernatants were aliquoted and frozen at -80°C, and thawed for single use. The same stocks of each virus were used for all experiments.

For experimental infection, MOI was approximated as the ratio of PFU to cells. Cells were exposed to a known titre of virus in a standard volume (50 μL for 96 well plates, 250 μL for 24 well plates, 1000 μL for 6 well plates) in macrophage or microglia medium. In parallel, cells were mock infected with medium in the same volume but without virus. At 2 hours post-infection, the inoculum was removed and replaced with fresh medium (macrophage or microglia), with or without treatments (IFNs, RUX etc) as indicated, until the time required for the experiment.

### Plaque assay

Supernatant from infected cell cultures were harvested and stored at the indicated time points. Aliquots of supernatant were thawed and serially diluted in DMEM with 1% FCS (Gibco), and 250 μL added to 24-well plates of confluent Vero cells. After 2 hours of incubation, 1.5% methylcellulose in DMEM with 1% FCS (Gibco) was gently added. At 72 hours post-infection, media was aspirated and cells fixed with 4% formaldehyde, before being stained with 0.25% crystal violet stain, washed and plaques counted on a lightbox to determine plaque-forming units per mL (PFU/mL).

### Quantitative RT-PCR

iPS-Mϕ were lysed in BL buffer at 24h.p.i. ZIKV^FP^ MOI=10, iPS-MGL were lysed in BL buffer at 24h.p.i. ZIKV^FP^ MOI=1. T otal RNA was extracted using the ReliaPrepTM RNA Cell Miniprep System (Promega, USA) and treated with DNase I according to the manufacturer’s instructions. Quantity of purified RNA was measured spectrophotometrically (A260/A280) using a NanoDrop One^C^ Microvolume UV-Vis Spectrophotometer (Thermo Fisher, MA, USA). RT-PCR was performed using the SensiFAST™ Probe No-ROX One-Step Kit (Bioline, USA) with AriaMx Real-time PCR System (Agilent Technologies, CA, USA) according to manufacturer’s instructions. The following TaqMan gene expression assay (Thermo Fisher) was used: *IFNA1* (Hs03044218_g1). The primers were designed using the Roche Universal ProbeLibrary Assay Design tool (Roche, Basel, Switzerland) with the indicated UPL probes. Further details, including additional primer/probe information, are summarised in **Table S1**. Target gene expression was normalised to the housekeeper *GAPDH* or *18S* as indicated. Each sample was run in technical duplicate. Cycling conditions were as follows: reverse transcription at 50°C for 15 min, followed by initial polymerase activation at 95°C for 10 min, and then 40 cycles of denaturation at 95°C for 15 sec and annealing/extension at 60°C for 1 min.

### RNA sequencing

RNA was extracted from iPS-Mϕ at 24h.p.i. ZIKV^FP^ MOI=10 using BL buffer and ReliaPrepTM RNA Cell Miniprep System (Promega) according to the manufacturer’s instructions. Sequencing was performed over four lanes of a single flow cell on an Illumina NextSeq (Illumina, USA) over 75 cycles. Sequencing was single ended. Raw FASTQ files were first inspected for quality using FastQC (69) and MultiQC (70). All FASTQ files were of a very high quality, so no filtering or trimming was performed. Pseudo-alignment and transcript counting was performed and generated for the four FASTQ files for each sample using Salmon (71). These counts are then imported into R for subsequent analysis. Transcripts were agglomerated into gene counts. Individual sample counts were merged into a single count table of samples as columns and genes as rows, with cells representing individual raw counts. Library normalisation, dispersion estimation, and log transformation was performed on the count table using the DESeq2 pipeline (72), set as default. RNA-seq data were uploaded to the Gene Expression Online (GEO) repository (accession no. GSE198542).

### Flow cytometry

Macrophages were lifted using EDTA and washed with FACS buffer (PBS plus 2% FCS and 0.05% sodium azide). Cells were stained at room temperature for one hour with the following antibodies: CD45 (clone 2D1, APC-H7), CD14 (clone M5E2, BUV737), CD11c (clone B-ly6, BV421), CD86 (clone 2331, FITC, all from BD), HLA-DR (clone L243, BV650), CD11b (clone ICRF4, BV785, all from Biolegend) or the respective isotype controls. Cell viability was assessed using 7-AAD (Biolegend). After the final washing step samples were acquired on a Symphony A5 flow cytometer (BD) and results were analysed using FlowJo (Ashland, OR, USA). See **Figure S2** for the gating strategy.

### Phagocytosis assay

IPS-macrophages were lifted using EDTA and resuspended in fresh macrophage medium (XVIVO15 containing 1% Penicillin/Streptomycin, 1% GlutaMAX, 100 ng/mL MCSF). pHrodo red *Zymosan A* bioparticles (Invitrogen) were diluted at a concentration of 100,000 beads per ul and mixed with the iPS-macrophages at a ratio of 10:1 beads to cells. Cells were either placed on ice immediately (negative control) or incubated for 2 hours at 37°C in a rocking incubator. Afterwards cells were immediately placed on ice to stop phagocytosis and 4′,6-diamidin-2-phenylindol (DAPI; ThermoFisher) was added to assess viability. Samples were acquired without further washing steps on a Symphony A5 flow cytometer (BD Biosciences) and results were analyzed using FlowJo (Ashland, OR, USA).

### Immunoblotting

Proteins from cell lysates were separated by 10% sodium dodecyl sulphate–polyacrylamide gel electrophoresis (SDS-PAGE) gel electrophoresis using MOPS running buffer (Thermo Fisher), and transferred to a nitrocellulose membrane (Millipore, USA) using NuPage Tris-Glycine Transfer Buffer (Thermo Fisher) for immunoblotting (antibodies see **Table S2**). Blots were developed with Pierce ECL Western blotting substrate (Thermo Fisher) and imaged on a LI-COR Odyssey Fc (LI-COR, NE, USA). Densitometry analysis was undertaken using ImageStudio software (version 5.2.5, Li-COR).

### Immunofluorescence

Cells were grown on eight-well chamber slides (Millipore). Following treatment and/or infection, cells were fixed with 4% paraformaldehyde in PBS for 20 minutes at room temperature before blocking/permeabilisation with 0.5% Triton X-100 (Sigma-Aldrich, USA)/10% goat serum (Abcam, UK) in PBS for 1 hour at room temperature. Cells were incubated with mouse anti-ZIKA ENV and rabbit anti-IFITM3 specific primary antibodies (see **Table S2**) overnight at 4°C, then washed three times in PBS and incubated with goat anti-mouse IgG Alexa fluor 488 or goat anti-rabbit IgG Alexa fluor 555 secondary antibody (both 1 μg/ml; Thermo Fisher) for 1 hour at RT. Nuclei were stained with 4′,6-diamidino-2-phenylindole (DAPI; 0.2 μg/ml; Sigma-Aldrich). All fluorescent images were taken using an EVOS FL fluorescence microscope (Thermo Fisher) at 10X magnification using DAPI, GFP, RFP and bright-field filters. Image analysis was performed using CellProfiler software version 3.1.8 (Broad institute, USA) (73).

Images were converted to greyscale, and within DAPI images each object (nucleus) was identified using the IdentifyPrimaryObject module. In order to quantify proteins within the whole cell including the cytoplasm, cells were identified within bright-field images by propagating out from each nucleus using the IdentifySecondaryObjects module, to create a mask for each cell. The intensity of the defined cells was measured and then predefined thresholds for GFP (Env protein) and RFP (IFITM3) were used to classify Zika positive and/or ISG productive cells, and the average value of at least n=4 images per well was used for analysis. A total of 58 sets of images (since all biological replicates were made up of at least n=3 technical replicate wells) with a median (IQR) of 255.5 (162 – 513.25) cells per image processed.

### Cell viability assay

Cells in 96 well plates were infected at the indicated MOI for 72 hours, with or without pre-treatment with the agents indicated for 16 hours, and imaged. Live cell imaging solution containing 3 drops/mL of ReadyProbes Cell Viability Blue/Red Imaging Kit (Invitrogen) was added 30 minutes before imaging to assess cell viability. Images were obtained using an EVOS FL fluorescence microscope (Thermo Fisher) using DAPI and RFP filters at 10x magnification. Image analysis was performed using CellProfiler version 3.1.8 (Broad institute, USA). DAPI and RFP images were converted to greyscale and objects identified using IdentifyPrimaryObject modules. DAPI objects (corresponding to Hoechst nuclear staining) overlapping with RFP objects (corresponding to propidium iodide staining) were classified as dead cells, while non-overlapping DAPI objects were counted as live cells. All conditions were performed in technical duplicate and the average value of n=6 images per well was used for analysis. All experiments were performed at least three times. The cell profiler pipelines used are available at https://github.com/aidanhanrath/Cellprofiler.

### Statistical analysis

Statistical analysis was performed using GraphPad Prism 9 software (GraphPad Software, CA, USA). Values are presented as mean ± SD of at least three independent experimental replicates. Continuous data were normalised or log-transformed prior to analysis using parametric significance tests. Statistical significances between two groups of data were determined using unpaired Student’s t-test and between three or more groups were determined using one-way analysis of variance (ANOVA), with Sidak’s *post-hoc* test to account for multiple comparisons. All tests were two-sided and an alpha of < 0.05 was the threshold for statistical significance.

## Data availability statement

Source data are provided with this paper as a data supplement. The source data file includes uncropped blots, all raw quantitative data and PFU counts. The results of differential expression analysis of RNA-seq data are also included as a supplementary dataset. Raw RNA seq data are available on GEO (Accession: GSE198542).

## Author contributions

AH, CH, FG, CB, JV, PL, SJC and CD did experiments and analysed data. SAC, WJ, SH and CD obtained funding and provided supervision. AH and CD conceived the study and with CH drafted the manuscript. All authors contributed to the article and approved the submitted version.

## Funding

AH was funded by an NIHR Academic Clinical Fellowship (ACF-2018-01-004) and the British Medical Association Foundation. FG is supported by the Munich Clinician Scientist Program at LMU (FoeFoLeplus) and received fellowships from the Bubble Foundation as well as the Care-for-Rare Foundation. CH and CD are funded by the UK Medical Research Council [MR/N013840/1 and MR/X001598/1 respectively]. SH and CD are funded by the Wellcome Trust [207556/Z/17/Z and 211153/Z/18/Z respectively]. iPS cell derivation and gene editing was carried out at the James Martin Stem Cell Facility, which receives financial support from the Oxford Martin School (LC0910-004).

## Acknowledgments

We are grateful to Jon Coxhead and Rafiq Ahmed for assistance with Illumina RNA-sequencing, and to Javier Gilbert Jaramillo for discussions.

## Conflict of interest

SH declares honoraria from CSL Behring and Takeda for teaching and consultancy.

The remaining authors declare that the research was conducted in the absence of any commercial or financial relationships that could be construed as a potential conflict of interest.

## Publisher’s note

All claims expressed in this article are solely those of the authors and do not necessarily represent those of their affiliated organizations, or those of the publisher, the editors and the reviewers. Any product that may be evaluated in this article, or claim that may be made by its manufacturer, is not guaranteed or endorsed by the publisher..
