# Supplementary material for: Type I interferon receptor (IFNAR2) deficiency reveals Zika virus cytopathicity in human macrophages and microglia
Source: Front Immunol. 2022 Nov 11;13:1035532. doi: 10.3389/fimmu.2022.1035532 (PMC9691778; doi:10.3389/fimmu.2022.1035532)
Supplement: Supplementary file 2 [file DataSheet_2.docx]

**Type I Interferon receptor (*IFNAR2*) deficiency reveals Zika virus cytopathicity in human macrophages and microglia**

**Extended data and supplementary material**

**Contents:**

Supplementary Tables S1 and S2

Supplementary Figures S1 and S2

See also source data file and supplementary dataset containing RNA-seq data (uploaded separately).

| **Gene** | **UPL probe** | **Forward sequence** | **Reverse sequence** |
| --- | --- | --- | --- |
| *IFNB* | #25 | CGACACTGTTCGTGTTGTCA | GAAGCACAACAGGAGAGCAA |
| *IFNL1* | #75 | GGGACCTGAGGCTTCTCC | CCAGGACCTTCAGCGTCA |
| *IL1B* | #78 | TACCTGTCCTGCGTGTTGAA | TCTTTGGGTAATTTTTGGGATCT |
| *IL6* | #40 | GATGAGTACAAAAGTCCTGATCCA | CTGCAGCCACTGGTTCTGT |
| *GAPDH* | #87 | TGGTATCGTGGAAGGACTCA | GCCATCACGCCACAGTTT |

**Supplementary Table S1. QRT-PCR primer/probe sequences.** Note *IFNA1* and *TNF* were assessed using TaqMan (Thermofisher) gene expression assays: *IFNA1* (Hs03044218_g1), *TNF* (Hs00174128_m1).

| **Antibody** | **Host** | **Working dilution** | **Source** | **Code** |
| --- | --- | --- | --- | --- |
| IFNAR2 | Sheep | 1:200 | R&D systems | AF7014 |
| IFITM3 | Rabbit | 1:10000 | ProteinTech | 11714-1-AP |
| RSAD2 | Rabbit | 1:1000 | CST | 13996 |
| ISG15 | Rabbit | 1:1000 | CST | 2743 |
| STAT2 | Mouse | 1:2000 | SCB | sc-1668 |
| pSTAT2 | Rabbit | 1:2000 | CST | 8841 |
| STAT1 | Rabbit | 1:1000 | CST | 9172 |
| pSTAT1 | Rabbit | 1:1000 | CST | 7649 |
| Tmem119 | Rabbit | 1:100 | Abcam | Ab185333 |
| Iba1 | Goat | 1:500 | Abcam | Ab5076 |
| Zika Envelope | Mouse | 1:5000 | BioFront Tech | BF-1176-56 |
| α-tubulin | Mouse | 1:10,000 | CST | 3873 |
| GAPDH | Rabbit | 1:10,000 | CST | 5174 |
| Anti-rabbit HRP- conjugated | Goat | Various | CST | 7074 |
| Anti-mouse HRP-conjugated | Horse | Various | CST | 7076 |

**Supplementary Table S2. Immunoblotting and immunofluorescence antibodies.** CST = Cell Signalling Technologies; HRP = horseradish peroxidase; SCB = Santa Cruz Biotechnology.

**Supplementary Figure**

**Figure S1. Preserved responses to IFNγ in *IFNAR2* deficient human iPS-macrophages.**

Immunoblot of pSTAT1, STAT1, IRF1 and GAPDH in IFNγ (1000 IU/mL) treated *IFNAR2*^PT^ (clone 6) and *IFNAR2*^WT^ (WT1) iPS-Mφ, representative of n = 3 independent experiments.


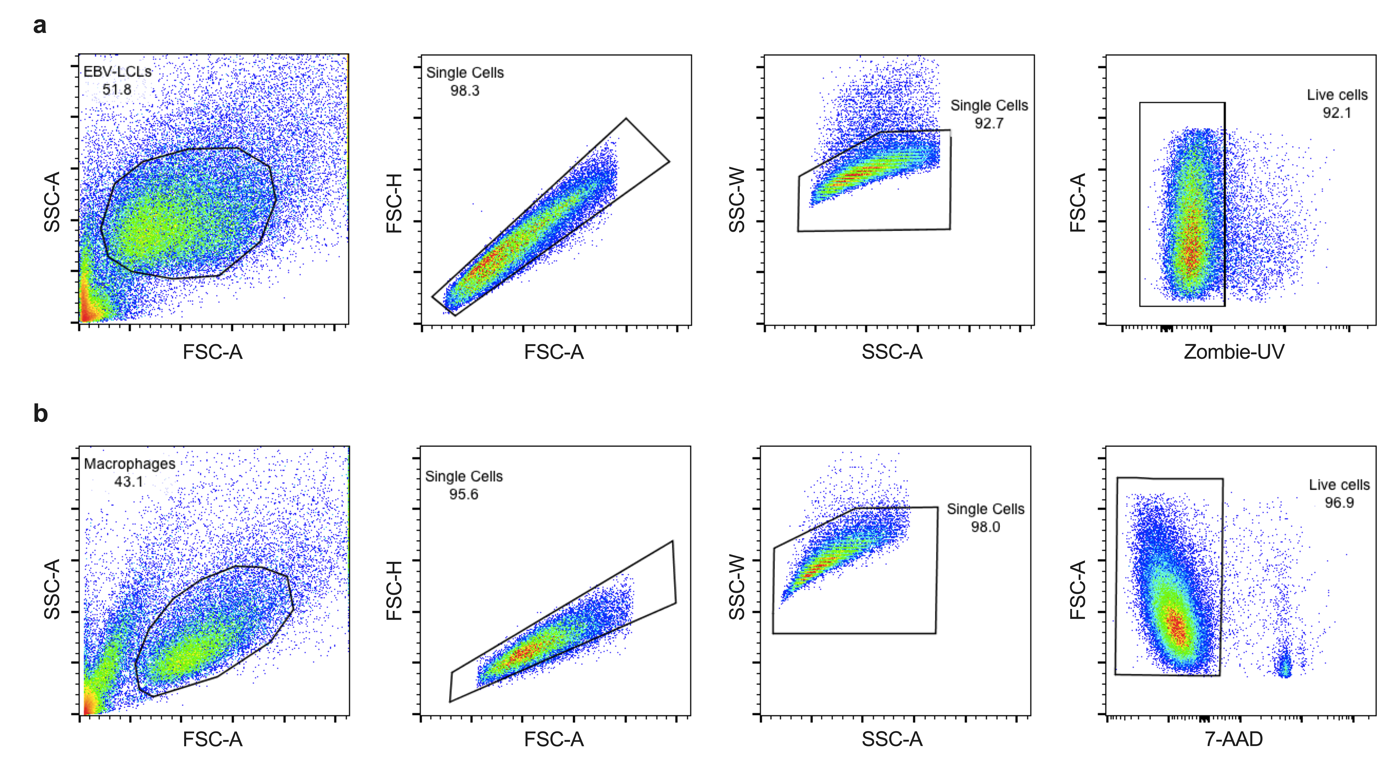


**Figure S2.** Representative gating strategy for flow cytometric analysis of iPS-macrophages.
